# Impact of Polyunsaturated Fatty Acids on miRNA Profiles of Monocytes/Macrophages and Endothelial Cells—A Pilot Study

**DOI:** 10.3390/ijms18020284

**Published:** 2017-01-28

**Authors:** Claudia Roessler, Kevin Kuhlmann, Christine Hellwing, Anja Leimert, Julia Schumann

**Affiliations:** Clinic for Anesthesiology and Surgical Intensive Care, University Hospital Halle (Saale), Franzosenweg 1a, 06112 Halle (Saale), Germany; claudia.roessler@uk-halle.de (C.R.); Big_Brainkuhlmann@web.de (K.K.); christine.hellwing@uk-halle.de (C.H.); anja.leimert@medizin.uni-halle.de (A.L.)

**Keywords:** miRNA expression, PUFA, membrane composition, macrophages, endothelial cells

## Abstract

Alteration of miRNAs and dietary polyunsaturated fatty acids (PUFAs) underlies vascular inflammation. PUFAs are known to be incorporated into the cell membrane of monocytes/macrophages or endothelial cells, the major cellular players of vascular diseases, thereby affecting cellular signal transduction. Nevertheless, there are no investigations concerning the PUFA impact on miRNA expression by these cells. With regard to the key role miRNAs play for overall cellular functionality, this study aims to elucidate whether PUFAs affect miRNA expression profiles. To this end, the monocyte/macrophage cell line RAW264.7 and the endothelial cell line TIME were enriched with either docosahexaenoic acid (DHA; n3-PUFA) or arachidonic acid (AA; n6-PUFA) until reaching a stable incorporation into the plasma membrane and, at least in part, exposed to an inflammatory milieu. Expressed miRNAs were determined by deep sequencing, and compared to unsupplemented/unstimulated controls. Data gained clearly show that PUFAs in fact modulate miRNA expression of both cell types analyzed regardless the presence/absence of an inflammatory stimulator. Moreover, certain miRNAs already linked to vascular inflammation were found to be affected by cellular PUFA enrichment. Hence, vascular inflammation appears to be influenced by dietary fatty acids, inter alia, via PUFA-mediated modulation of the type and amount of miRNAs synthesized by cells involved in the inflammatory process.

## 1. Introduction

Fine-tuning of protein expression is of great significance for overall cellular functionality. Gene transcription and translation not only need to be adjusted to meet the demands of the single eukaryotic cell but also to ensure an optimum interaction in higher multi-cellular organisms. Dysregulation of these biological processes are frequently associated with disease development and progression.

Non-coding RNAs, in particular microRNAs (miRNAs), play an important role in gene regulation. Preponderantly, miRNAs are able to inhibit the expression of a certain protein by means of translation repression or even mRNA degradation, but some miRNAs even can stimulate translation [1,2,3]. Each of these short, endogenous, regulatory RNAs can target a wide variety of gene transcripts due to imperfect base pairing, thereby mediating post-transcriptional gene silencing [1,2,3,4]. What is more, a particular mRNA can be co-regulated by several miRNAs resulting in a complex, interconnected, and coordinated regulatory network [1,2,4]. Consequently, aberrant processes and a large number of disease states including inflammatory disorders and cardiovascular conditions have been implicated with changes in miRNA expression [1,3]. Illnesses in general and inflammatory cascades in particular are characterized by the mutual interaction of various cellular players, each baring its own miRNA signature. For example, vascular inflammation, which is a hallmark of atherosclerosis as well as sepsis, is believed to originate from the interplay between activated monocytes and endothelial cells [5,6,7]. Hence, a prerequisite for in-depth understanding of vascular inflammation is the analysis of disease-associated alterations in miRNA expression in a cell type-specific manner.

It is well accepted that cellular miRNA synthesis depends on the surrounding microenvironment. This implicates that blood-borne cells, such as monocytes, as well as cells with constant blood contact, such as endothelial cells, are influenced in their miRNA profiles by blood composition. This, in turn, is modulated, besides other factors, by nutritional components, for instance dietary polyunsaturated fatty acids (PUFAs). PUFAs are known to affect inflammatory reactions and are reported to play a role in vascular dysfunction [8,9]. Epidemiologic and interventional studies concurrently link PUFA supplementation with protective effects on adverse cardiovascular events, a reduction in arterial stiffness and vascular inflammation, and even clinical improvements in sepsis patients [8,10,11,12,13]. Laboratory investigations indicate that endotoxin-induced production of pro-inflammatory cytokines, such as TNF-α, IL-1β, and IL-6, is reduced in PUFA-enriched monocytes [11,14,15]. Further on, endothelial expression of membrane-bound as well as soluble adhesion molecules is reduced due to PUFA administration [12,16]. The PUFA-mediated decrease of vascular inflammation goes along with an improvement of endothelial function [13,16]. What is more, dietary balance in fatty acid intake influences leukocyte-endothelial interactions. For example, monocyte rolling, adhesion, and trans-endothelial migration are described to be affected by PUFAs [17]. Based on the health effects of PUFAs, various nutritional societies, e.g., the European Society for Clinical Nutrition and Metabolism (ESPEN), encourage the use of PUFA-enriched lipid emulsions instead of pure saturated fatty acid-based lipid emulsion in intensive care. The mechanisms underlying the PUFA effects on monocyte and endothelial cell functionality, however, are not well understood. So far, the following potential general modes of action have been described: (1) conversion of PUFA into eicosanoids or resolvins, which are potent immune-regulators; (2) the function of PUFAs as ligands for the immune cell receptors peroxisome proliferator-activated receptor gamma (PPARγ) or G-protein coupled receptor 120 (GPR120); and (3) PUFA-mediated modulation of plasma membrane and lipid raft composition, thereby influencing cellular signal transduction [9,18,19]. In addition, there are indications from feeding studies that PUFAs might impact cellular miRNA signatures [20,21]. The data on this, however, are surprisingly poor. To our knowledge in scientific literature, there is no information concerning the impact of PUFAs on miRNA expression of monocytes/macrophages or endothelial cells.

The present study aims to fill this knowledge gap. For this purpose, monocytes/macrophages and endothelial cells were supplemented with PUFAs in a physiological relevant concentration [22], and subsequently exposed to an inflammatory milieu. Resulting alterations in miRNA profiles were analyzed by deep sequencing. Non-supplemented as well as non-stimulated cells served as reference. The data gained provide evidence that PUFAs in fact modulate cellular miRNA profiles. The PUFA impact on cellular miRNA expression was observed for both cell types tested and regardless of the presence of an inflammatory state. Since measured effects were found for cells demonstrably PUFA-enriched in their cell membrane [23,24], we propose that changes in cellular membrane fatty acid composition affects signal transduction, which results in an altered transcription of miRNAs.

## 2. Results

In this study, miRNA expression profiles of the monocyte/macrophage cell line RAW264.7 as well as the endothelial cell line TIME were analyzed. Up- or downregulated miRNAs were identified due to (i) an inflammatory milieu; (ii) PUFA supplementation; or (iii) a combination of inflammatory milieu and PUFA supplementation. Expression levels of a miRNA were considered when there was at least a two-fold disparity between the comparison groups (*n* = 3 for each subgroup).

### 2.1. Monocyte/Macrophage Cell Line RAW264.7

#### 2.1.1. Impact of Lipopolysaccharide (LPS) Stimulation

Following exposure to LPS 57 miRNAs were identified to be alternatively expressed in stimulated RAW264.7 compared to control cells. These included 18 upregulated miRNAs, and 22 downregulated miRNAs (Table 1). Furthermore, 11 miRNAs could only be identified in LPS-treated RAW264.7, whereas 6 miRNAs could not be detected in the LPS in contrast to the control group (Table 1).

#### 2.1.2. Impact of PUFA Supplementation

Enrichment of RAW264.7 with the n3-PUFA docosahexaenoic acid (DHA) resulted in the alternative expression of 29 miRNAs. Compared to unsupplemented control cells 6 miRNAs were upregulated, and 16 miRNAs were downregulated in RAW264.7 cultured in DHA-enriched medium with downregulation attaining a fold-change of 0.2 for 2 of these miRNAs (Table 2). Furthermore, one miRNA could be identified in DHA treated RAW264.7 only (Table 2). A total of six miRNAs could be detected in control cells but not in cells enriched in DHA (Table 2).

Supplementation of RAW264.7 with the n6-PUFA arachidonic acid (AA) resulted in the alternative expression of 29 miRNAs. Compared to unsupplemented control cells, seven miRNAs were upregulated, and eight miRNAs were downregulated in RAW264.7 cultured in AA enriched medium (Table 3). Seven miRNAs could only be identified in AA treated RAW264.7, and a further seven miRNAs could not be detected in the AA in contrast to the control group (Table 3).

**Figure 1:**
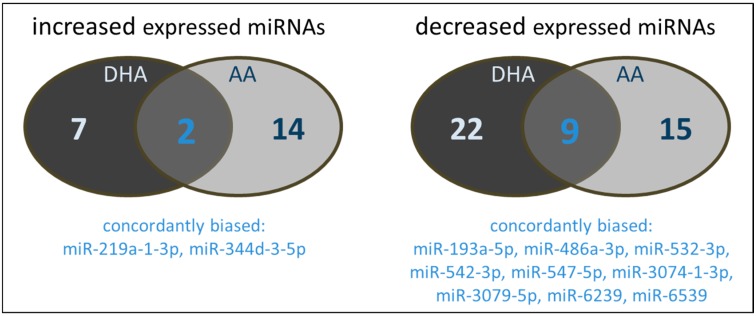
Number and type of miRNAs of polyunsaturated fatty acids (PUFA) supplemented (72 h, 15 µM) RAW264.7 modulated in their expression by either docosahexaenoic acid (DHA) and/or arachidonic acid (AA).

It has to be noted that there are distinct differences in the miRNAs influenced by the PUFAs tested. As shown in Figure 1a, a certain proportion of miRNAs modulated by DHA was not affected by AA and vice versa. Only two miRNAs were increased by both DHA and AA. From the miRNAs characterized by a decreased expression profile, nine were concordantly modulated by both DHA and AA.

#### 2.1.3. Impact of a Combination of LPS Stimulation and PUFA Supplementation

LPS stimulation of RAW264.7 previously enriched with the n3-PUFA DHA resulted in the alternative expression of 50 miRNAs. Compared to control cells, 5 miRNAs were upregulated, and 32 miRNAs were downregulated in DHA-supplemented RAW264.7 triggered with LPS (Table 4). Of these, five miRNAs showed an expression reduction of at least five-fold. A total of 3 miRNAs were detected in RAW264.7 treated with DHA plus LPS but not in the untreated control group, whereas 10 miRNAs could be identified in control cells only (Table 4).

Combined treatment of RAW264.7 with LPS plus the n6-PUFA AA resulted in the alternative expression of 86 miRNAs. Compared to the control group, 8 miRNAs were upregulated, and 59 miRNAs were downregulated in RAW264.7 enriched in AA and stimulated with LPS (Table 5). For 13 of these miRNAs, downregulation attained a fold-change of at least 0.2. In particular, miR-143-3p was characterized by an expression reduction by a factor of 500. There was one miRNA that could only be identified in LPS-triggered RAW264.7 cultivated in AA supplemented medium (Table 5). In addition, a total of 18 miRNAs could solely be detected in untreated control cells but not in RAW264.7 after combined treatment using LPS and AA (Table 5).

**Figure 2:**
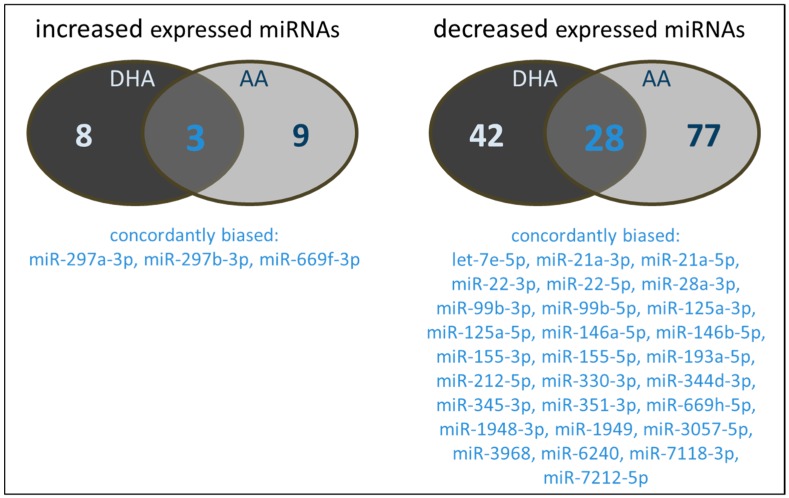
Number and type of miRNAs of PUFA supplemented (72 h, 15 µM) and LPS stimulated (24 h, 1 µg/mL) RAW264.7 modulated in their expression by either DHA and/or AA.

Again, there are distinct differences in the miRNAs influenced by the PUFA tested (Figure 2). Three miRNAs were found to be increased by both DHA and AA. Regarding downregulated miRNAs, a total of 28 were concordantly modulated by both DHA and AA. Of note, there is almost no overlap between the miRNAs alternatively expressed in RAW264.7 treated with a combination of PUFA supplementation and LPS stimulation and the miRNAs up- or downregulated in RAW264.7 exclusively treated with either PUFA or LPS.

### 2.2. Endothelial Cell Line TIME

#### 2.2.1. Impact of Cytokine Stimulation

Following exposure to the pro-inflammatory cytokines IL-1β, TNF-α, and IFN-γ 173 miRNAs were identified to be alternatively expressed in stimulated TIME, compared to control cells. These included 151 upregulated miRNAs and 4 downregulated miRNAs (Table 6). Of these, 20 miRNAs showed differences greater than five-fold. Furthermore, 18 miRNAs could only be identified in cytokine treated TIME but not in the unstimulated control group (Table 6). There was no disappearance of miRNAs due to cytokine stimulation.

#### 2.2.2. Impact of PUFA Supplementation

Enrichment of TIME with the n3-PUFA DHA resulted in the alternative expression of 21 miRNAs. Compared to unsupplemented control cells, 16 miRNAs were upregulated, and one miRNA was downregulated in TIME cultured in DHA-enriched medium (Table 7). Three miRNAs could be identified in DHA-treated TIME only (Table 7). Furthermore, one miRNA could be detected in control cells but not in cells enriched in DHA (Table 7).

Supplementation of TIME with the n6-PUFA AA resulted in the alternative expression of 140 miRNAs. Compared to unsupplemented control cells, 131 miRNAs were upregulated in TIME cultured in AA enriched medium with three miRNAs being increased five-fold and beyond (Table 8). Moreover, nine miRNAs were solely detectable in AA treated TIME (Table 8). AA supplementation did not result in a downregulation or disappearance of any miRNAs.

**Figure 3:**
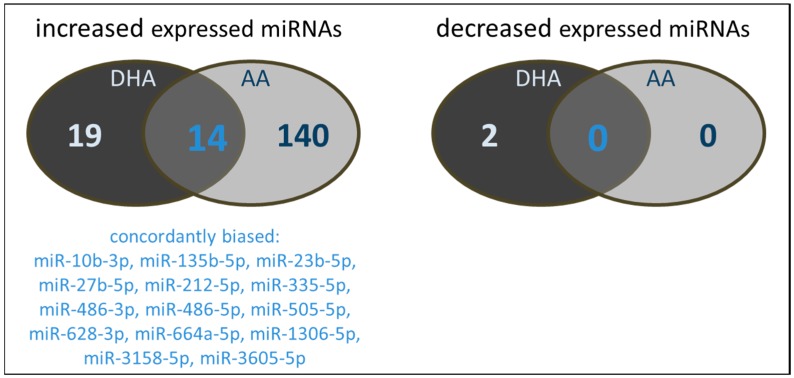
Number and type of miRNAs of PUFA supplemented (144 h, 15 µM) TIME modulated in their expression by either DHA and/or AA.

The number of miRNAs concurrently influenced by both DHA and AA is shown in Figure 3. Despite the great differences in the absolute numbers of affected miRNAs, there is a considerable degree of convergence in the nature of miRNAs biased by PUFA enrichment in general. From the miRNAs characterized by an increased expression profile, a total of 14 were concordantly modulated by both DHA and AA.

#### 2.2.3. Impact of a Combination of Cytokine Stimulation and PUFA Supplementation

Combined treatment of TIME with cytokines plus the n3-PUFA DHA resulted in the alternative expression of 46 miRNAs. Compared to control cells, four miRNAs were upregulated, and 31 miRNAs were downregulated in DHA-supplemented TIME triggered with the cytokines IL-1β, TNF-α, and IFN-γ (Table 9). Of these, the miRNA miR-1-3p showed an expression increase of about 118-fold. Beyond that, two miRNAs were detected in TIME treated with DHA plus cytokines but not in the untreated control group (Table 9). Nine miRNAs could solely be identified in control cells (Table 9).

Cytokine stimulation of TIME previously enriched with the n6-PUFA AA resulted in the alternative expression of 16 miRNAs. Compared to the control group, three miRNAs were upregulated, and eight miRNAs were downregulated in TIME enriched in AA and stimulated with IL-1β and TNF-α plus IFN-γ (Table 10). There was one miRNA, which could only be identified in cytokine-triggered TIME cultivated in AA supplemented medium (Table 10). In addition, a total of four miRNAs could solely be detected in untreated control cells but not in TIME after combined treatment using cytokines and AA (Table 10).

**Figure 4:**
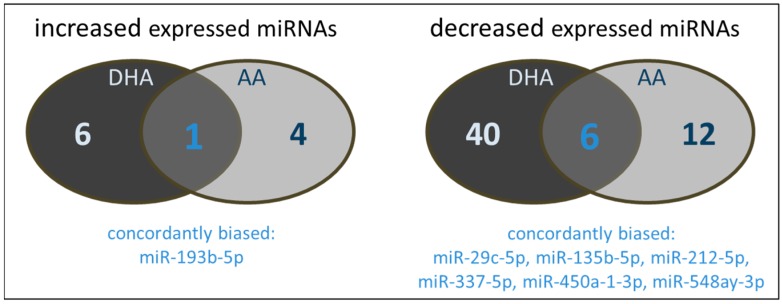
Number and type of miRNAs of PUFA supplemented (144 h, 15 µM) and cytokine stimulated (IL-1β + TNF-α + IFN-γ, 24 h, 5 ng/mL each) TIME modulated in their expression by either DHA and/or AA.

There are distinct differences in the miRNAs influenced by either DHA or AA. The majority of miRNAs modulated by DHA was not affected by AA and vice versa (Figure 4). Only one miRNA was increased by both DHA and AA. From the miRNAs characterized by a decreased expression profile, six were concordantly modulated by both DHA and AA. As seen for the monocyte/macrophage cell line RAW264.7, there is no or almost no overlap between the miRNAs alternatively expressed in endothelial cells treated with a combination of PUFA supplementation and cytokine stimulation and the miRNAs up- or downregulated in endothelial cells exclusively treated with either PUFA or cytokines.

### 2.3. In Silico Analysis

**Figure 5:**
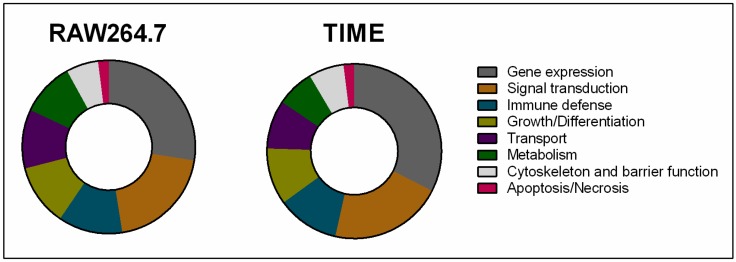
In silico clustering of genes targeted by PUFA-modulated miRNAs to basic biological processes.

Identified PUFA-regulated miRNAs were subjected to an in silico analysis by means of the miRWalk 2.0 database in order to determine previously validated corresponding target genes. The Database for Annotation, Visualization, and Integrated Discovery (DAVID) functional annotation clustering tool was used to perform a KEGG pathway and GO term enrichment analysis on tagged miRNA target genes. Next, Kyoto Encyclopedia of Genes and Genoms- (KEGG) and Gene Ontology (GO)-related clusters were related to basic biological processes, namely metabolism, signal transduction, growth/differentiation, apoptosis/necrosis, gene expression, cytoskeleton, and barrier function, transport, as well as immune defense. It was found that major targets of miRNAs alternatively expressed in PUFA-enriched cells are linked to gene expression and signal transduction without tangible differences between the cell types or inflammatory conditions analyzed (Figure 5). This was followed by immune defense-, growth/differentiation-, and transport-associated genes (Figure 5).

Key miRNAs that are particularly altered by PUFAs and validated to play a role in inflammation are displayed in Table 11 for the monocyte/macrophage cell line RAW264.7 and in Table 12 for the endothelial cell line TIME. For both cell lines investigated, there are a wide variety of target genes and thus immunological processes under the regulatory control of PUFA-affected miRNAs. It should be noted that several miRNAs biased by PUFA supplementation have concurring target genes. An outstanding number of immunological relevant target genes were found for hsa-miR-335-5p, which in TIME cells is modulated by supplementation of both DHA and AA.

## 3. Discussion

Here we present the first analysis of the PUFA impact on monocytes/macrophage and endothelial cell miRNA expression profiles in either a normal or an inflammatory milieu. For both cell types, our data provide evidence that PUFA enrichment affects the kind and the amount of particular miRNAs synthesized. It should be noted that, in our study, cellular miRNA profiles were measured as a function of long-term PUFA supplementation, which leads to an incorporation of the fatty acids into the plasma membrane. The periods of supplementation used in our experiments are already proved by us to result in a membrane fatty acid steady state [23,24]. From this perspective, our data hint toward an innovative mechanism of PUFA action. It seems plausible that there is a causal link between the plasma membrane lipid composition and the miRNA expression of a cell.

So far, studies concerning the interrelation between PUFA and miRNA expression are limited, concentrating primarily on tumor cell biology [25,26]. Information on monocytes/macrophages or endothelial cells is missing although these cells are in constant blood contact, making them particularly susceptible to nutrition-based influences. Both cell types are directly involved in inflammatory processes of the vascular system [5,6,7] and in turn have been said to be influenced by unsaturated fatty acids [8,9]. Inflammatory states of the vasculature have been correlated to both miRNA expression [1,3] and fatty acid supply; nevertheless, miRNA expression and fatty acid supplyhave not yet been related. With the recognition that there is a direct connection between the dietary PUFA content and the plasma membrane lipid composition of monocytes/macrophages and endothelial cells on the one hand, and the miRNA expression of these cells on the other hand, our study points the way to a new understanding of the PUFA-mediated modulation of vascular inflammatory processes.

With a view to the literature, certain miRNAs have already been linked to vascular inflammation, whether they are in acute inflammatory states as in the case of sepsis or chronic inflammatory conditions as in the case of atherosclerosis. Several of these miRNAs also pop up in our study in case of LPS-stimulated RAW264.7 or cytokine-stimulated TIME, which supports the validity of the data set. Beyond that, PUFA supplementation itself demonstrably impacts these inflammation-associated miRNAs; however, it turns out that there are distinct differences in PUFA effects between the cell types tested. Looking into the details, an upregulation of miR-10a, miR-17-3p, miR-125a, miR-155, and miR-181b was observed due to AA supplementation of endothelial cells in the absence of an inflammatory stimulus. All of these miRNAs are described to possess anti-inflammatory actions, attenuating the expression of adhesion molecules and inflammation-driving cytokines [27,28]. DHA in turn was observed to downregulate miR-146a in the context of an inflammatory milieu. This miRNA is known to contribute to the induction of vascular inflammation [29]. It can therefore be stated that, by this means, both DHA and AA seem to support an anti-inflammatory endothelial state. With regard to the monocytes/macrophages tested, the PUFA-mediated impact on proven inflammation-associated miRNAs is even more pronounced. Under inflammatory conditions, both DHA and AA downregulate numerous miRNAs, which are well-known for their involvement in Toll-like receptor signaling and macrophage differentiation. Namely expression of miR-21, miR-125a, miR146a, miR-146b, and miR-155, which are all described as miRNA targets of TLR signaling [27,28,30], are decreased due to RAW264.7 enrichment with either DHA or AA. PUFAs, therefore, may be seen as modulators of macrophage phenotype and inflammatory response. This is in accordance with our previously published functional analyses demonstrating PUFAs such as DHA or AA to impact virtually all macrophage features including cytokine synthesis, respiratory burst, phagocytosis, and the expression of adhesion molecules [15,31,32].

Besides these literature-described miRNAs, DAVID functional annotation clustering indicates that there are numerous further PUFA-targeted miRNAs, which are linked to immune defense. About 10% of all miRNAs alternatively expressed in either RAW264.7 or TIME due to PUFA enrichment belong in this group, thus opening an exciting field in vascular inflammation research. For example, miR-1-3p, whose expression is increased by a factor of 118 in cytokine-stimulated, DHA-supplemented endothelial cells, may influence the interplay between cytokines and their corresponding receptors, and is predicted to interact with various members of the Jak-STAT signaling pathway.

Comparing the PUFAs analyzed, it is found that there are distinct differences in the miRNA targets of the fatty acids. It is interesting to note that, beside the deviations observed for particular miRNAs, there seems to be a common focus, as can be seen in the context of the literature-described miRNAs discussed above.

## 4. Materials and Methods

### 4.1. Materials

All chemicals and reagents were obtained from Sigma-Aldrich (Taufkirchen, Germany) unless noted otherwise. Cell culture flasks were purchased from Greiner Bio-One (Frickenhausen, Germany). HEPES (25 mmol/L)-buffered RPMI 1640 culture medium containing 300 mg/L l-glutamine was acquired from Pan-Biotech (Aidenbach, Germany). Microvascular endothelial growth medium as per customer specification according to ATCC recommendations was acquired from Provitro AG (Berlin, Germany).

### 4.2. Cell Culture, Fatty Acid Supplementation, and Stimulation

The mouse monocyte/macrophage cell line RAW264.7 (ATCC number: TIB-71) as well as the human telomerase-immortalized microvascular endothelial cell line TIME (ATCC number: CRL-4025) were used. RAW264.7 were cultured in RPMI 1640 medium containing 4.5 g/L glucose, 5% *v*/*v* FCS, and 0.2% *v*/*v* ethanol (basic medium). TIME was cultured in basal microvascular endothelial growth medium enriched with 5 ng/mL VEGF, 5 ng/mL EGF, 5 ng/mL FGF, 15 ng/mL IGF-1, 10 mM l-glutamine, 0.75 U/mL heparin sulfate, 1 µg/mL hydrocortisone hemisuccinate, 50 µg/mL ascorbic acid, 5% *v*/*v* FCS, 12.5 µg/mL blasticidin, and 0.2% *v*/*v* ethanol (basic medium). The fatty acids docosahexaenoic acid (DHA, C22:6n3) or arachidonic acid (AA, C20:4n6) (all Biotrend, Köln, Germany) were included in the culture medium in concentrations of 15 µmol/L using ethanol as a vehicle (0.2% *v*/*v* final ethanol concentration). Cells were cultured in the enriched media in 75 cm^2^ cell culture flasks totaling either 72 h (RAW264.7) or 144 h (TIME) at 37 °C and 5% CO_2_ in a humidified atmosphere. Stimulation of cells was performed in the last 24 h of fatty acid supplementation by an addition of either LPS (1 µg/mL; from E. coli serotype 0111:B4) for cell line RAW264.7 or the cytokines IL-1β, TNF-α, and IFN-γ each in a concentration of 5 ng/mL (all PeproTech, Hamburg, Germany) for cell line TIME. Periods of supplementation and stimulation were proven to result in a membrane fatty acid steady state as well as reproducible effects on macrophage/endothelial cell functionality [15,23,24,31,32].

### 4.3. RNA Isolation, Deep Sequencing, and Analysis of Deep Sequencing Data

Total RNA extraction was performed using a standard liquid–liquid extraction protocol based on TRIzol LS (Thermo Fisher Scientific, Dreieich, Germany) according to the manufacturer’s instructions. Quality of RNA gained was analyzed by means of the NanoDrop spectrophotometer (Thermo Fisher Scientific, Dreieich, Germany) as well as the Agilent Bioanalyzer (Agilent Technologies, Waldbronn, Germany). Analysis of miRNA expression by high-throughput sequencing was performed in the Core Unit DNA, Leipzig University by means of a Illumina HiScanSQ (Illumina Inc., San Diego, CA, USA). Briefly, samples were prepared using the TruSeq Small RNA Prep kit v2 (Illumina Inc., San Diego, CA, USA) according to the manufacturer’s instructions. The barcoded libraries were size restricted (140–165 bp), purified, and quantified using the Library quantification kit—Illumina/Universal (KAPA Biosystems, Woburn, MA, USA) according to the manufacturer’s instructions. A pool of up to 10 libraries was used for cluster generation per lane using an Illumina cBot. Sequencing of 50 bp was performed with an Illumina HighScanSQ sequencer using version 3 chemistry and flowcell according to the manufacturer’s instructions. For analysis of the deep sequencing data, adapter sequences were removed from raw sequences using Cutadapt software [33]. From the remaining sequences, only those 15–27 bases long were kept for further analysis. Alignment of these reads to murine/human genome as well as mature sequences of miRBase v21 was done using the bowtie aligner [34]. Data were compressed with Samtools [35] to bam format. Mapped reads were counted with R/Bioconductor programming environment [36] by application of the ShortRead library [37]. An error rate of 1 nt per mature miRNA sequence was allowed. Normalization of data was performed by the two independent procedures DESeq2 and the TMM algorithm.

### 4.4. Statistical Analysis

Two-way analysis of variance followed by unpaired Student’s *t*-test was used to identify significant differences between means. The statistical analysis was carried out by means of the program GraphPad Prism 6 (GraphPad Software, La Jolla, CA, USA). In all cases, *p* < 0.05 was considered to indicate significant differences. Significantly differentially expressed miRNAs, which had a sequence read >10 and were regulated greater than 2-fold, were depicted in the data tables.

### 4.5. miRNA Target Prediction

In silico identification of miRNA targets was performed by importing the list of alternatively expressed miRNAs into the miRWalk 2.0 database [38,39] (http://zmf.umm.uni-heidelberg.de/apps/zmf/mirwalk2/index.html), which combines twelve prediction datasets (miRWalk, miRDB, PITA, MicroT4, miRMap, RNA22, miRanda, miRNAMap, RNAhybrid, miRBridge, PICTAR2, and Targetscan). For identification of pathway distribution of validated targets as well as enrichment of these genes for functional categories the Database for Annotation, Visualization, and Integrated Discovery (DAVID) 6.8 Beta [40,41] (https://david-d.ncifcrf.gov) was used, which utilizes the Kyoto Encyclopedia of Genes and Genomes (KEGG) database and Gene Ontology (GO) terms.

## 5. Conclusions

As this is a pilot study, details regarding expression levels of individual miRNAs should be taken with caution. Clearly, a validation of data, for example, by means of a TaqMan-based polymerase chain reaction, should be performed for miRNAs of interest. Nevertheless, with regard to the data presented here, the following conclusions can be drawn: (1) PUFAs impact the miRNA profiles of monocytes/macrophages and endothelial cells; (2) PUFA-mediated alteration of membrane micro-domains modulate cellular signal transduction, thereby influencing the transcription of non-coding RNAs such as miRNAs; (3) in doing so, PUFAs are able to affect vascular inflammation in a miRNA-based manner. Altogether, this study leads the way to a new understanding of PUFA-mediated cellular signal transduction in general and, in particular, of the dietary modulation of inflammatory processes taking place in the vascular system.

## Figures and Tables

**Table 1:** Significantly differentially expressed miRNAs (sequence read >10; regulated greater than 2-fold) in RAW264.7 due to LPS stimulation (24 h, 1 µg/mL).

Fold-Change	miRNAs
3.6	miR-505-5p
3.2	miR-126a-3p, miR-126b-5p
3.0	miR-33-5p, miR-199b-3p, miR-223-3p, miR-466h-3p, miR-1943-5p
2.7	miR-3057-5p
2.5	miR-199a-3p
2.3	miR-8114
2.2	miR-151-5p, miR-182-5p
2.1	miR-183-5p
2.0	miR-181a-2-3p, miR-212-5p, miR-450a-1-3p, mmiR-3065-3p
0.3	miR-32-3p, miR-130b-3p, miR-201-5p, miR-497a-5p, miR-6539
0.4	miR-32-5p, miR-129-5p, miR-148a-3p, miR-148b-3p, miR-350-3p, miR-466c-5p, miR-542-3p, miR-1191a, miR-3963
0.5	miR-125b-2-3p, miR-148b-5p, miR-224-5p, miR-297a-3p, miR-301a-3p, miR-301a-5p, miR-450b-5p, miR6239
solely detected in control	let-7f-1-3p, miR-1198-3p, miR-331-3p, miR-547-5p, miR-3079-5p, miR-146a-3p
solely detected in experimental	miR-143-3p, miR-145a-3p, miR-145a-5p, miR-6240, miR-451a, miR-351-3p, miR-345-3p, miR-1b-5p, miR-1a-3p, miR-7118-3p, miR-7212-5p

**Table 2:** Significantly differentially expressed miRNAs (sequence read >10; regulated greater than 2-fold) in RAW264.7 due to DHA supplementation (72 h, 15 µM).

Fold-Change	miRNAs
2.8	miR-344d-3-5p
2.6	miR-129b-3p
2.4	miR-219a-1-3p
2.2	miR-210-3p, miR-1943-5p
2.0	miR-450a-1-3p
0.2	miR-139-3p, miR-183-5p
0.4	let-7f-1-3p, miR-182-5p, miR-200b-3p, miR-542-3p, miR-574-3p, miR-3074-1-3p
0.5	miR-25-3p, miR-30d-5p, miR-193a-5p, miR-365-3p, miR-486a-3p, miR-669c-5p, miR-1306-5p, miR-1949
solely detected in control	miR-193a-5p, miR-532-3p, miR-547-5p, miR-3079-5p, miR-6239, miR-6539
solely detected in experimental	miR-375-3p

**Table 3:** Significantly differentially expressed miRNAs (sequence read >10; regulated greater than 2-fold) in RAW264.7 due to AA supplementation (72 h, 15 µM).

Fold-Change	miRNAs
4.0	miR-505-5p
3.8	miR-3057-5p
2.7	miR-669h-5p
2.4	miR-344d-3-5p
2.3	miR-8114
2.2	miR-219a-1-3p
2.0	miR-378a-5p
0.4	miR-29c-3p, miR-146a-3p, miR-486a-3p, miR-542-3p, miR-669p-3p
0.5	miR-155-3p, miR-669f-3p, miR-3074-1-3p
solely detected in control	miR-193a-5p, miR-331-3p, miR-532-3p, miR-547-5p, miR-3079-5p, miR-6239, miR-6539
solely detected in experimental	miR-1a-3p, miR-26b-3p, miR-96-5p, miR-125b-1-3p, miR-143-3p, miR-185-3p, miR-345-3p

**Table 4:** Significantly differentially expressed miRNAs (sequence read >10; regulated greater than 2-fold) in RAW264.7 due to combined DHA supplementation (72 h, 15 µM) and LPS stimulation (24 h, 1 µg/mL).

Fold-Change	miRNAs
3.3	miR-297a-3p
2.6	miR-297c-3p
2.5	miR-669f-3p
2.2	miR-200a-3p
2.1	miR-1b-5p
0.1	miR-155-5p, miR-344d-3p
0.2	miR-125a-3p, miR-139-3p, miR-193a-5p
0.3	let-7e-5p, miR-28a-3p, miR-92a-1-5p, miR-99b-3p, miR-146b-5p, miR-181a-1-3p, miR-582-3p, miR-1949, miR-3057-5p, miR-3968
0.4	miR-7b-5p, miR-21a-5p, miR-28a-5p, miR-33-5p, miR-92a-3p, miR-99b-5p, miR-125a-5p, miR-146a-5p, miR-222-3p, miR-330-3p, miR-339-3p
0.5	miR-21a-3p, miR-22-3p, miR-22-5p, miR-221a-5p, miR-664-5p, miR-669h-5p
solely detected in control	miR-155-3p, miR-212-5p, miR-345-3p, miR-351-3p, miR-1943-5p, miR-1948-3p, miR-6240, miR-6537-3p, miR-7118-3p, miR-7212-5p
solely detected in experimental	miR-297b-3p, miR-935, miR-3064-5p

**Table 5:** Significantly differentially expressed miRNAs (sequence read > 10; regulated greater than 2-fold) in RAW264.7 due to combined AA supplementation (72 h, 15 µM) and LPS stimulation (24 h, 1 µg/mL).

Fold-Change	miRNAs
3.9	miR-497a-5p
3.6	miR-30b-3p
2.6	miR-466c-5p
2.5	miR-342-3p, miR-669a-3p, miR-669f-3p
2.3	miR-297a-3p
2.0	miR-201-5p
0.002	miR-143-3p
0.1	miR-21a-3p, miR-155-5p, miR-344d-3p
0.2	miR-10b-5p, miR-21a-5p, miR-125a-3p, miR-126a-3p, miR-126a-5p, miR-126b-5p, miR-146b-5p, miR-199b-3p, miR-877-5p
0.3	let-7e-5p, miR-27a-3p, miR-28a-3p, miR-99b-3p, miR-125a-5p, miR-146a-5p, miR-199a-3p, miR-450b-3p, miR-466h-3p, miR-486b-3p, miR-1949, miR-1983, miR-3057-5p
0.4	let-7b-5p, let-7c-2-3p, let-7d-3p, miR-16-1-3p, miR-22-3p, miR-22-5p, miR-25-5p, miR-26b-5p, miR-99b-5p, miR-101b-3p, miR-130b-5p, miR-193a-5p, miR-196a-5p, miR-330-3p, miR-330-5p, miR-486a-3p, miR-505-5p, miR-547-3p, miR-652-3p, miR-877-3p, miR-1843b-5p, miR-3968
0.5	let-7a-1-3p, let-7a-5p, let-7b-3p, miR-7a-5p, miR-27a-3p, miR-151-5p, miR-365-3p, miR-450b-5p, miR-486b-5p, miR-574-3p, miR-669h-5p
solely detected in control	miR-1a-3p, miR-1b-5p, miR-29b-2-5p, miR-145a-3p, miR-145a-5p, miR-155-3p, miR-199a-5p, miR-212-5p, miR-345-3p, miR-351-3p, miR-451a, miR-532-3p, miR-872-3p, miR-1948-3p, miR-6240, miR-6537-3p, miR-7118-3p, miR-7212-5p
solely detected in experimental	miR-297b-3p

**Table 6:** Significantly differentially expressed miRNAs (sequence read >10; regulated greater than 2-fold) in TIME due to cytokine stimulation (24 h, IL-1β + TNF-α + IFN-γ 5 ng/mL each).

Fold-Change	miRNAs
16.1	miR-195-3p
15.2	miR-29b-1-5p
12.5	miR-92a-1-5p
10.9	miR-23a-5p
9.0	miR-27a-5p, miR-92b-5p
7.5	miR-1275, miR-3615
6.7	miR-320c
6.4	miR-155-5p
5.9	miR-17-3p, miR-4455
5.7	miR-1185-2-3p, miR-4443
5.5	miR-146b-5p, miR-320b
5.3	miR-29b-3p, miR-320d
5.2	miR-212-5p
5.1	miR-4521
4.6	miR-135b-5p
4.4	miR-455-5p, miR-629-5p
4.3	miR-21-3p, miR-130a-3p, miR-151b, miR-193a-5p
4.2	miR-29b-2-5p
4.1	miR-26a-2-3p, miR-125a-3p, miR-503-5p, miR-505-5p, miR-2110
4.0	miR-1226-3p
3.9	miR-29a-3p, miR-181b-5p, miR-299-3p, miR-486-3p, miR-655-3p
3.8	miR-125b-1-3p, miR-186-5p
3.7	miR-25-5p
3.6	miR-221-5p, miR-1180-3p, miR-486-5p
3.5	miR-27a-3p, miR-146a-5p, miR-2355-3p
3.4	miR-22-5p, miR-4451
3.3	miR-10b-3p, miR-192-5p, miR-312-5p, miR-331-5p
3.2	miR-432-5p
3.1	miR-181b-3p, miR-377-3p
3.0	miR-24-2-5p, miR-27b-3p, miR-193b-3p, miR-320a, miR-423-5p, miR-548k, miR-576-3p, miR-1306-5p, miR-1307-3p
2.9	miR-181a-5p, miR-195-5p, miR-574-5p, miR-3184-3p
2.8	let-7b-5p, miR-221-3p, miR-382-5p, miR-485-5p, miR-3130-5p, miR-3158-5p
2.7	miR-484
2.6	miR-361-3p, miR-543, miR-652-3p, miR-760, miR-3120-3p
2.5	miR-16-2-3p, miR-495-3p, miR-1262, miR-1255a
2.4	miR-27b-5p, miR-30e-3p, miR-127-5p, miR-130b-3p, miR-140-3p, miR-181c-5p, miR-199a-3p, miR-199b-3p, miR-214-5p, miR-224-3p, miR-410-3p, miR-433-3p, miR-450a-1-3p, miR-450a-5p, miR-487a-3p, miR-497-5p, miR-744-5p, miR-942-5p, miR-1261, miR-3613-3p, miR-4677-3p
2.3	let-7a-2-3p, miR-7-1-3p, miR-16-5p, miR-24-3p, miR-101-3p, miR-106b-3p, miR-381-3p, miR-3074-5p, miR-3182, miR-4448
2.2	let-7d-5p, miR-21-5p, miR-134-5p, miR-326, miR-328-3p, miR-337-5p, miR-431-5p, miR-589-5p, miR-628-3p, miR-1301-3p, miR-4326
2.1	miR-7-5p, miR-15b-3p, miR-32-5p, miR-98-3p, miR-98-5p, miR-106b-5p, miR-126-5p, miR-151a-5p, miR-154-3p, miR-224-5p, miR-335-5p, miR-450b-5p, miR-493-5p, miR-548e-3p, miR-576-5p, miR-651-5p, miR-664a-5p, miR-3940-3p
2.0	let-7g-5p, miR-92b-3p, miR-214-3p, miR-411-5p, miR-665
0.3	let-7c-3p
0.4	miR-380-3p, miR-671-3p
0.5	miR-654-3p
solely detected in experimental	miR-29a-5p, miR-29c-5p, miR-30b-3p, miR-146a-3p, miR-155-3p, miR-330-5p, miR-376b-3p, miR-487a-5p, miR-496, miR-539-5p, miR-548ay-3p, miR-877-5p, miR-1254, miR-1908-5p, miR-3130-3p, miR-3158-3p, miR-3614-5p, miR-4662a-5p

**Table 7:** Significantly differentially expressed miRNAs (sequence read >10; regulated greater than 2-fold) in TIME due to DHA supplementation (72 h, 15 µM).

Fold-Change	miRNAs
4.0	miR-628-3p
3.3	miR-1306-5p
3.1	miR-505-5p
2.8	miR-486-3p
2.7	miR-486-5p, miR-664a-5p
2.6	miR-23b-5p, miR-27b-5p
2.5	miR-212-5p
2.4	miR-320d
2.3	miR-328-3p, miR-1180-3p, miR-3605-3p
2.1	miR-10b-3p, miR-335-5p
2.0	miR-135b-5p
0.5	miR-1271-5p
solely detected in control	miR-1185-1-3p
solely detected in experimental	miR-9-5p, miR-3177-3p, miR-3158-3p

**Table 8:** Significantly differentially expressed miRNAs (sequence read >10; regulated greater than 2-fold) in TIME due to AA supplementation (72 h, 15 µM).

Fold-Change	miRNAs
5.8	miR-27b-5p, miR-628-3p
5.0	miR-23b-5p
4.3	miR-486-3p, miR-486-5p
4.2	miR-412-5p
4.1	miR-182-5p
3.8	miR-181b-3p
3.5	miR-195-3p, miR-3605-3p
3.4	miR-505-5p
3.3	miR-139-3p, miR-216a-3p, miR-1271-5p
3.2	miR-655-3p, miR-7706
3.1	miR-212-5p, miR-628-5p
3.0	miR-423-5p, miR-584-5p
2.9	miR-10b-3p, miR-379-3p, miR-432-5p, miR-548e-3p, miR-1180-3p, miR-1304-3p, miR-1306-5p
2.8	miR-128-3p, miR-191-3p, miR-361-3p, miR-362-3p, miR-664a-3p, miR-1275, miR-3074-3p, miR-3173-5p
2.7	let7e-3p, miR-26a-2-3p, miR-98-3p, miR-125b-2-3p, miR-135b-5p, miR-374a-3p, miR-450b-5p, miR-485-5p, miR-3615, miR-4677-3p
2.6	let7e-5p, miR-17-3p, miR-92b-5p, miR-99b-3p, miR-130b-5p, miR-143-3p, miR-323a-3p, miR-874-3p, miR-4510
2.5	miR-10a-5p, miR-23c, miR-26b-3p, miR-30a-5p, miR-30d-5p, miR-98-5p, miR-99b-5p, miR-151a-3p, miR-487a-3p, miR-493-5p, miR-532-5p, miR-676-3p, miR-3184-3p
2.4	let7b-5p, let7c-5p, let7d-3p, miR-30c-5p, miR-100-3p, miR-155-5p, miR-335-5p, miR-424-3p, miR-455-5p, miR-500a-3p, miR-769-5p, miR-1255a, miR-3130-5p
2.3	miR-10b-5p, miR-106b-3p, miR-125a-5p, miR-342-3p, miR-382-3p, miR-484, miR-502-3p, miR-548k, miR-1226-3p
2.2	let7a-5p, miR-25-3p, miR-25-5p, miR-30e-3p, miR-92a-3p, miR-224-5p, miR-320c, miR-340-3p, miR-409-3p, miR-454-3p, miR-501-3p, miR-532-3p, miR-543, miR-1301-3p, miR-3529-3p
2.1	let7d-5p, let7g-5p, miR-7-5p, miR-26a-5p, miR-127-5p, miR-192-5p, miR-320b, miR-340-5p, miR-362-5p, miR-421, miR-454-5p, miR-574-5p, miR-651-5p, miR-664a-5p, miR-1307-3p
2.0	miR-23a-3p, miR-28-3p, miR-148b-3p, miR-181a-2-3p, miR-199a-3p, miR-320a, miR-410-3p, miR-629-5p, miR-769-5p, miR-889-3p, miR-2110, miR-2682-5p
solely detected in experimental	miR-30b-3p, miR-145-3p, miR-183-5p, miR-216a-5p, miR-539-5p, miR-548o-3p, miR-3130-3p, miR-3158-3p, miR-4662a-5p

**Table 9:** Significantly differentially expressed miRNAs (sequence read >10; regulated greater than 2-fold) in TIME due to combined DHA supplementation (72 h, 15 µM) and cytokine stimulation (24 h, IL-1β + TNF-α + IFN-γ 5 ng/mL each).

Fold-Change	miRNAs
118.0	miR-1-3p
3.2	miR-193b-5p
2.6	miR-216a-5p
2.2	miR-3605-3p
0.3	miR-29c-5p, miR-140-5p, miR-212-5p, miR-374b-3p, miR-576-5p, miR-582-3p
0.4	miR-15b-3p, miR-19a-3p, miR-337-5p, miR-376a-3p, miR-410-3p, miR-487a-3p, miR-487a-5p, miR-505-5p, miR-628-5p, miR-889-3p
0.5	miR-24-2-5p, miR-26a-2-3p, miR-30c-5p, miR-135b-5p, miR-195-5p, miR-338-3p, miR-381-3p, miR-454-3p, miR-497-5p, miR-539-3p, miR-589-5p, miR-655-3p, miR-4521, miR-4662a-5p, miR-4677-3p
solely detected in control	miR-136-5p, miR-146a-3p, miR-200c-3p, miR-377-5p, miR-450a-1-3p, miR-548ay-3p, miR-641, miR-1255a, miR-3152-5p
solely detected in experimental	miR-4731-3p, miR-548l

**Table 10:** Significantly differentially expressed miRNAs (sequence read >10; regulated greater than 2-fold) in TIME due to combined AA supplementation (72 h, 15 µM) and cytokine stimulation (24 h, IL-1β + TNF-α + IFN-γ 5 ng/mL each).

Fold-Change	miRNAs
2.5	miR-450a-2-3p
2.4	miR-193b-5p, miR-204-3p
0.4	miR-224-3p, miR-1304-3p
0.5	miR-7-1-3p, miR-29c-5p, miR-135b-5p, miR-212-5p, miR-337-5p, miR-3615
solely detected in control	miR-370-5p, miR-450a-1-3p, miR-548ay-3p, miR-3158-3p
solely detected in experimental	miR-589-3p

**Table 11:** Validated immune defense-associated target genes of miRNAs differentially expressed in RAW264.7 due to either PUFA supplementation (DHA or AA, 72 h, 15 µM) or combined PUFA supplementation plus LPS stimulation (24 h, 1 µg/mL) plus.

miRNAs	Affected by	Target Genes	
		Name	Entrez ID
miR-1a-3p	LPS, AA AA + LPS	*Adenosine deaminase, RNA-specific (Adar)*	56417
		*E26 avian leukemia oncogene 1, 5′ domain (Ets1)*	23871
miR-1b-5p	LPS, DHA + LPS, AA + LPS	*CD28 antigen (Cd28)*	12487
miR-10b-5p	AA + LPS	*A kinase (PRKA) anchor protein 8 (Akap8)*	56399
		*DNA cross-link repair 1C (Dclre1c)*	227525
		*E26 avian leukemia oncogene 1, 5′ domain (Ets1)*	23871
		*Fas (TNFRSF6)-associated via death domain (Fadd)*	14082
miR-21a-5p	DHA + LPS, AA + LPS	*Fas ligand (Fasl)*	14103
miR-30d-5p	DHA	*CD300A molecule (Cd300a)*	217303
		*Calcium-dependent protein kinase IV (Camk4)*	12326
		*Colony stimulating factor 1 (macrophage) (Csf1)*	12977
		*Interleukin 18 receptor 1 (Il18r1)*	16182
miR-125a-3p	DHA + LPS, AA + LPS	*TNF receptor-associated factor 6 (Traf6)*	22034
		*V-set immunoregulatory receptor (Vsir)*	74048
		*Transcription complex subunit NF-ATc4 (Nfatc4)*	73181
		*Signal transducer activator of transcription 1 (Stat1)*	20846
miR-126a-3p	LPS, DHA + LPS	*POU domain, class 2, transcription factor 2 (Pou2f2)*	18987
		*SAM domain and HD domain, 1 (SAMHD1)*	56045
miR-146a-5p	DHA + LPS, AA + LPS	*TNF receptor-associated factor 6 (Traf6)*	23034
		*Interferon gamma (Ifng)*	15978
		*Interleukin-1 receptor-associated kinase 1 (Irak1)*	16179
		*Interleukin-1 receptor-associated kinase 2 (Irak2)*	108960
miR-146b-5p	DHA + LPS	*Nuclear factor kappaB p50 (Nfkb1)*	18033
miR-155-5p	DHA + LPS, AA + LPS	*MHC class I-related gene protein (Mr1)*	15064
		*E26 avian leukemia oncogene 1, 5′ domain (Ets1)*	23871
		*Fas (TNFRSF6)-associated via death domain (Fadd)*	14082
		*Inhibitor of kappaB kinase epsilon (Ikbke)*	56489
miR-199a-5p	AA + LPS	*CD4 antigen (Cd4)*	12504
miR-210-3p	DHA	*Inositol polyphosphate-5-phosphatase D (Inpp5d)*	16331
		*Lymphocyte cytosolic protein 2 (lcp2)*	16822
		*Neural cell adhesion molecule 1 (Ncam1)*	17967
miR-222-3p	DHA + LPS	*5-azacytidine induced gene 2 (Azi2)*	27215
miR-297b-3p	DHA + LPS, AA + LPS	*2′-5′ oligoadenylate synthetase 3 (Oas3)*	246727
		*CD28 antigen (Cd28)*	12487
miR-345-3p	LPS, AA, DHA + LPS, AA + LPS	*SLAM family member 8 (Slamf8)*	74748
miR-365-3p	DHA	*Macrophage MHC class I receptor 2 (Mfsd6)*	98682
miR-375-3p	DHA	*Janus kinase 2 (Jak2)*	16452
		*Complement component 1 binding protein (C1qbp)*	12261
		*Attractin (Atrn)*	11990
miR-466h-3p	LPS, AA + LPS	*Fas (TNFRSF6)-associated via death domain (Fadd)*	14082
miR-542-3p	LPS, DHA, AA	*CD1d1 antigen (Cd1d1)*	12479
miR-582-3p	DHA + LPS	*CD300 molecule like family member F (Cd300lf)*	246746
miR-669a-3p	AA + LPS	*CD47 antigen (Cd47)*	16423
miR-669c-5p	DHA	*Fyn proto-oncogene (Fyn)*	14360
		*Chemokine (C-X-C motif) ligand 5 (Cxcl5)*	20311
miR-669f-3p	AA, DHA + LPS, AA + LPS	*2'-5' oligoadenylate synthetase 3 (Oas3)*	246727
		*CD200 receptor 1 (Cd200r1)*	57781
		*Butyrophilin-like 9 (Btln9)*	237754
		*Interleukin 17 receptor A (Il17ra)*	16172
		*Interleukin 5 receptor, α (Il5rα)*	16192
		*Lymphocyte antigen 96 (Ly96)*	17087
		*Melanoma cell adhesion molecule (Mcam)*	84004
		*Paired-Ig-like receptor A1 (Pira1)*	18722
		*Tec protein tyrosine kinase (Tec)*	21682
		*Selenoprotein S (Selenos)*	109815
miR-1943-5p	LPS, DHA, DHA + LPS	*TLR4 interactor with leucine-rich repeats (Tril)*	66873
miR-3064-5p	DHA + LPS	*AXL receptor tyrosine kinase (Axl)*	26362
		*CD300A molecule (Cd300a)*	217303
miR-3074-1-3p	DHA	*Leukocyte surface antigen CD47 (Cd47)*	16423
miR-3079-5p	LPS, DHA	*Fas (TNFRSF6)-associated via death domain (Fadd)*	14082

**Table 12:** Validated immune defense-associated target genes of miRNAs differentially expressed in TIME due to PUFA supplementation (DHA or AA, 144 h, 15 µM) or combined PUFA supplementation plus cytokine stimulation (24 h, IL-1β + TNF-α + IFN-γ 5 ng/mL each).

miRNAs	Affected by	Target Genes	
		Name	Entrez ID
let-7b-5p	AA	*CD 81 molecule (Cd81)*	975
		*Immunoglobulin superfamily member 3 (IGSF3)*	3321
		*Immunoglobulin superfamily member 8 (IGSF8)*	93185
		*Interferon beta 1 (IFNB1)*	3456
		*Toll like receptor 4 (TLR4)*	7099
		*Neighbor of Punc E11 (IGDCC4)*	57722
miR-7-5p	Cytokines, AA	*Immunoglobulin superfamily member 8 (IGSF8)*	93185
		*Interleukin 21 receptor (IL21R)*	50615
		*Toll like receptor 4 (TLR4)*	7099
		*Immunoglobulin superfamily member 3 (IGSF3)*	3321
miR-9-5p	DHA	*CD200 molecule (CD200)*	4345
		*Immunoglobulin superfamily member 6 (IGFS6)*	10261
		*Interferon regulatory factor 1 (IRF1)*	3659
		*Interleukin5 (IL5)*	3567
miR-10a-5p	AA	*AXL receptor tyrosine kinase (AXL)*	558
		*Complement C5 (C5)*	727
		*Cytokine receptor like factor 3 (CRLF3)*	51379
		*Immunoglobulin superfamily member 1 (IGSF1)*	3547
		*Protein turtle homolog B (IGSFM9B)*	22997
		*Immunoglobulin-like transcript 1 (LILRA2)*	11027
miR-10b-5p	AA	*Beta-2-microglobulin (B2M)*	567
		*Cytokine receptor like factor 3 (CRLF3)*	51379
		*Immunoglobulin superfamily member 1 (IGSF1)*	3547
		*Immunoglobulin-like transcript 1 (LILRA2)*	11027
miR-17-3p	Cytokines, AA	*TNF α induced protein 1 (TNFAIP1)*	7126
miR-19a-3p	DHA+Cytokines	*CD22 molecule (CD22)*	933
		*Cytotoxic TRAIL receptor-2 (TNFRSF10B)*	8795
		*Receptor activator of NF-KB (TNFRSF11A)*	8792
		*TNF receptor superfamily member 1B (TNFRSF1B)*	7133
		*Toll like receptor 2 (TLR2)*	7097
miR-25-3p	AA	*Fas ligand (FASLG)*	356
		*Immunoglobulin superfamily member 1 (IGSF1)*	3547
		*Lymphocyte transmembrane adaptor 1 (LAX1)*	54900
		*B cell-activating factor receptor (TNFRSF13C)*	115650
		*Toll like receptor 3 (TLR3)*	7098
miR-30a-5p	AA	*Interferon epsilon (IFNE)*	338376
		*Interleukin 1 α (IL1A)*	3552
		*Cytotoxic TRAIL receptor-2 (TNFRSF10B)*	8795
miR-30c-5p	AA, DHA+Cytokines AA	*Interferon epsilon (IFNE)*	338376
		*Interleukin 1 α (IL1A)*	3552
		*Interleukin 11 (IL11)*	3589
		*Cytotoxic TRAIL receptor-2 (TNFRSF10B)*	8795
miR-30d-5p	Cytokines, AA	*Interferon epsilon (IFNE)*	338376
		*Interleukin 1 α (IL1A)*	3552
		*Cytotoxic TRAIL receptor-2 (TNFRSF10B)*	8795
miR-92b-3p	Cytokines, AA	*Fas ligand (FASLG)*	356
		*Toll like receptor 3 (TLR3)*	7098
		*Immunoglobulin superfamily member 8 (IGSF8)*	93185
miR-98-5p	AA	*Interleukin 6 (IL6)*	3569
		*Interleukin 13 (IL13)*	3596
		*Interleukin 32 (IL32)*	9235
		*TNF receptor superfamily member 9 (TNFRSF9)*	3604
		*CD46 molecule (CD46)*	4179
		*Complement C3 (C3)*	718
miR-125a-5p	AA	*CD244 molecule (CD244)*	51744
		*Interferon gamma (IFNG)*	3458
		*Interleukin 1 receptor antagonist (IL1RN)*	3557
		*Cytotoxic TRAIL receptor-2 (TNFRSF10B)*	8795
miR-130b-5p	AA	*CD84 molecule (CD84)*	8832
		*CD86 molecule (CD86)*	942
		*CD200 molecule (CD200)*	4345
		*Protein turtle homolog B (IGSDM9B)*	22997
miR-148b-3p	Cytokines, AA	*CD300a molecule (CD300A)*	11314
		*Immunoglobulin superfamily member 1 (IGSF1)*	3547
		*Immunoglobulin superfamily member 10 (IGSF10)*	285313
		*Interleukin 23 receptor (ILR23)*	149233
miR-155-5p	AA	*AXL receptor tyrosine kinase (AXL)*	558
		*Interleukin 2 (IL2)*	3558
		*Interleukin 6 (IL6)*	3569
		*Toll like receptor adaptor molecule 2 (TICAM2)*	353376
		*CD36 molecule*	948
		*CD81 molecule*	975
		*CD109 molecule (CD109)*	135228
		*Myeloid differentiation prim. response (MYD88)*	4615
miR-181a-2-3p	Cytokines, AA	*Interleukin 17 receptor E like (IL15REL)*	400935
		*Leukocyte-associated Ig-like receptor 1 (LAIR1)*	3903
		*Tumor necrosis factor receptor 13B (TNFRSF13B)*	23495
miR-192-5p	AA	*CD83 molecule (CD83)*	9308
		*Interleukin 7 (IL7)*	3574
		*Interleukin 15 (IL15)*	3600
		*Toll like receptor adaptor molecule 2 (TICAM2)*	353376
miR-216a-5p	Cytokines, DHA, AA	*CD84 molecule (CD84)*	8832
		*Complement C3 (C3)*	718
		*Interleukin 23 receptor (IL23R)*	149233
miR-335-5p	AA	*CD1d molecule (CD1d)*	912
		*CD8a molecule (CD8a)*	925
		*CD14 molecule (CD14)*	929
		*CD22 molecule (CD22)*	933
		*CD27 molecule (CD27)*	939
		*CD33 molecule (CD33)*	945
		*CD36 molecule (CD36)*	948
		*CD37 molecule (CD37)*	951
		*CD46 molecule (CD46)*	4179
		*CD79a molecule (CD79a)*	973
		*CD96 molecule (CD96)*	10225
		*CD101 molecule (CD101)*	9398
		*CD160 molecule (CD160)*	11126
		*CD177 molecule (CD177)*	57126
		*CD226 molecule (CD226)*	10666
		*CD276 molecule (CD276)*	80381
		*Interleukin 1 α (IL1A)*	3552
		*Interleukin 4 (IL4)*	3565
		*Interleukin 5 (IL5)*	3567
		*Interleukin 6 (IL6)*	3569
		*Interleukin 7 (IL7)*	3574
		*Interleukin 17a (IL17A)*	3605
		*Interleukin 22 (IL22)*	50616
		*Interleukin 25 (IL25)*	64806
		*Interleukin 27 (IL27)*	246778
		*Interleukin 33 (IL33)*	90865
		*Interferon α 21 (IFNA21)*	3452
		*G antigen 2C (GAGE2C)*	2574
		*G antigen 2D (GAGE2D)*	729408
		*G antigen 2E (GAGE2E)*	26749
		*G antigen 12B (GAGE12B)*	729428
		*G antigen 12D (GAGE12D)*	100132399
		*G antigen 12E (GAGE12E)*	729431
		*G antigen 12H (GAGE12H)*	729442
		*Toll like receptor 1 (TLR1)*	7096
		*Toll like receptor 2 (TLR2)*	7097
		*Toll like receptor 4 (TLR4)*	7099
		*Interleukin 17 receptor D (IL17RD)*	54756
		*Interleukin 21 receptor (IL21R)*	50615
		*F11 receptor (F11R)*	50848
		*TNF receptor superfamily member 9 (TNFRSF9)*	3604
		*Cytokine like 1 (CYTL1)*	54360
		*IgLON family member 5 (IGLON5)*	402665
		*Immunoglobulin superfamily member 10 (IGSF10)*	285313
miR-340-5p	AA	*Beta-2-microglobulin (B2M)*	567
		*CD84 molecule (CD84)*	8832
		*Immunoglobulin superfamily member 11 (IGSF11)*	152404
		*TRAIL receptor 1 (TNFRSF10A)*	8797
		*Cytotoxic TRAIL receptor-2 (TNFRSF10B)*	8795
miR-362-3p	DHA+Cytokines	*Complement C6 (C6)*	729
		*Cytokine receptor like factor 1 (CRLF1)*	9244
		*Fas ligand (FASLG)*	356
		*Leucocyte Ig-like receptor B1 (LILRB1)*	10859
		*leucocyte Ig-like receptor B2 (LILRB2)*	10288
		*B cell-activating factor receptor (TNFRSF13C)*	115650
		*TNFR-related death receptor 6 (TNFRSF21)*	27242
miR-377-5p	Cytokines, AA	*Complement C3 (C3)*	718
		*Cytokine receptor like factor 3 (CRLF3)*	51379
		*Interferon lambda receptor 1 (IFNLR1)*	163702
		*TNF receptor associated factor 1 (TRAF1)*	7189
miR-423-5p	Cytokines, AA	*Toll like receptor 5 (TLR5)*	7100
		*CD81 molecule (CD81)*	975
		*Neighbor of Punc E11 (IGDCC4)*	57722
miR-450a-1-3p	Cytokines, DHA+Cytokines, AA+Cytokines	*Immunoglobulin superfamily member 6 (IGSF6)*	10261
		*Polymeric immunoglobulin receptor (PIGR)*	5284
		*CD3d molecule (CD3D)*	915
miR-454-3p	AA, DHA+Cytokines	*Interferon regulatory factor 1 (IRF1)*	3659
		*Interleukin 23 receptor (IL23R)*	149233
		*Immunoglobulin-like transcript 1 (LILRA2)*	11027
		*Cytotoxic TRAIL receptor-2 (TNFRSF10B)*	8795
		*Interleukin 5 (IL5)*	3567
miR-485-5p	Cytokines, AA	*CMRF35-like molecule 9 (CD300LG)*	146894
		*CD4 molecule (CD4)*	920
		*Complement C3 (C3)*	718
		*G antigen 1 (GAGE1)*	2543
miR-574-5p	Cytokines, AA	*TNF α induced protein 8 (TNFAIP8)*	25816
		*CD28 molecule*	940
		*IgLON family member 5 (IGLON5)*	402665
miR-769-5p	AA	*Immunoglobulin superfamily member 3 (IGSF3)*	3321
		*Interleukin 17 receptor E like (IL17REL)*	400935
		*CDC4-like protein (LRBA)*	987
miR-1304-3p	AA, AA+Cytokines	*CD1 molecule (CD1)*	912
		*Complement C3 (C3)*	718
		*Immunoglobulin superfamily member 6 (IGSF6)*	10261
		*Immunoglobulin-like transcript 1 (LILRA2)*	11027
		*Lymphocyte transmembrane adaptor 1 (LAX1)*	54900
		*Tumor necrosis factor receptor 13B (TNFRSF13B)*	23495
